# Experimental Researches upon Febrile Caloricity, Both before and after Death—Post-mortem Fever

**Published:** 1844-06

**Authors:** Bennet Dowler

**Affiliations:** New Orleans


### WESTERN JOURNAL

OF

### MEDICINE AND SURGERY.

JUNE, 1844.

### Art. I.—*Experimental Researches upon Febrile Caloricity, both before and after death—Post-mortem Fever.* By Bennet Dowler, M.D., of New Orleans.

In these researches, it is not intended to enter upon the general theory of fever, though a few brief explanatory notices of some elementary, or rather incidental points, are deemed necessary for the purpose of illustration and comparison.

It has been often remarked, that the ancient fathers in medicine regarded fever in a very literal sense, as a morbid heat; a notion which, though ridiculed by some modern authors, has prevailed in all times, and in all places among the people. This simple view of fever will suffer little by comparison with the vague, cumbrous definitions of the most fastidious nosologists, such as Cullen, Good, and others. Dr. Billing, a late writer, says that the essential symptom of fever is "debility"—"all superadditions non-essential," (Prin. Med. p. 231 et seq.); Boisseau calls it an idiopathic local irritation, (Fevers, p. 490): Broussais holds the same doctrine. British authors, less simple in their definitions, give HEAT an obscure position in the catalogue. Wilson Philip, (vol. 1, p. 43, Fevers), makes fevers to consist in the following symptoms: "Languor, lassitude, and other signs of debility, followed by a frequent pulse and increased heat, without any primary local affection." Fordyce says, "Fever is a disease of the whole system," (Fevers, p. 16, et passim). Some authors regard putridity, some a hurried pulse, &c., as the essential characteristic, but after all the countless works upon the subject it would seem that no single symptom is more to be relied on than that given by Hippocrates and Galen,—a *burning*, a *morbid heat:* the latter considers that this *unnatural heat* originates in the heart, whence it is diffused over the whole body: "*Calor quidam præter naturam in corde generatus:*"—both these authors appear to regard febrile heat not only as to its augmentations, but also with respect to alterations of quality and unequal distributions in the body.

The ancients, it must be confessed, were sometimes very incorrect and extravagant in their estimates of the importance of heat. "*Quod calidum vocamus*," said Hippocrates, "*id mihi et immortale esse videtur, et cuncta intelligere, et videre, et audire, et scire omnia, tum præsentia tum futura.* (*Lib. de carn. c.* 1).

Multitudinous analogies might, perhaps, be traced between febrile caloricity, and concentrations of caloric applied to the body in burns of a certain degree; the effects are somewhat similar, as the rigor, the reaction, and even the internal lesions. Mr. Curling noticed vomitings and purgings of *blood*, and found acute ulcerations of the intestinal mucous tissue, chiefly in the duodenum, as results of burns. (Med. Chir. Rev., Jan. 1843). Baron Dupuytren gives a summary of the symptoms of burns in the first degree, over a large surface, in the following words: "The pulse rises, becomes frequent, the tongue reddens, and the phenomena of gastro-intestinal irritation are developed." (Leçons. 16). It will yet in all probability be proved even in algid or congestive fever, that the heat of the central parts is, what the patient feels it to be, very great, morbid, unnatural, characteristic.

As the following experiments and observations afford, in many instances, deductions so opposite to those of all the authors I have seen, I must throw myself upon the indulgence of the reader until all my facts have been given. And, since I intend to give the details of each experiment, so that others may prove or disprove them, I may without arrogance ask this much in advance. For example, if my observations show that there is an increase of heat after apparent death, a true *post-mortem fever*, hotter than is ever known during life—that the thigh is hotter than the epigastrum, the heart, the lungs, and the brain,—let not the reader conclude that I am mistaken, simply because others advance doctrines, not facts, quite different. Be it remembered that my observations contradict their opinions only, not their experiments, because they have made none, so far as I know, under the same circumstances. The animal heat is supposed to be greater in the brain, heart, lungs and stomach, than in the rectum or thigh, because the latter are farther from the centre. Now, so far as I have experimented, immediately, as well as some hours after death from fever, the contrary is generally true, and, in the absence of conflicting experiments, I think that no theoretical view can weigh a feather against facts.

I may here remark, that except the primary stage of fever, the best period, or *point de départ*, for ascertaining the true morbid heat proper to that malady is that immediately after death.

The temperature of the human body, after death from fever at least falls not under the dominion of the atmospheric temperature, in general, for a period more or less prolonged; on the contrary it augments. This fact appears conclusive against the exclusive respiratory or chemical origin of animal heat. Chemistry teaches, as it appears to me, a very different doctrine from that which makes the lungs a heating furnace—the calorific focus of the whole body. Ought not the abundance of water and carbon which take the gaseous form in the respiratory act, to cause a perpetual refrigeration to go on in the lungs during life? Transpiration by the skin, or evaporation, performs a similar function. Both these organs may act too much or too little; they may refrigerate too far below the standard of health, or they may fail to reduce a morbid heat when once kindled up. Vitalism presents antagonistic barriers more or less effectual to these functional deviations. Cutaneous and pulmonary transpiration, chemistry and vitality, may thus combine, during life, to lessen the most intense morbid heat of fever, as well as in cases of external heat. But in the moment of death, this triune coalition is broken—the vital forces are scattered—cutaneous and pulmonary refrigeration is overthrown, and for an indefinite period, often short, the morbid heat, the true fever, un-repressed, reveals itself, until dissipated by the antagonism of the surrounding temperature. Heat and cold are no more produced, augmented, or diminished, except by those laws which govern inert matter alone.

Perspiration, a chemico-vital action necessary to life, showing itself often as sweat, but still oftener eluding our vision as an invisible vapor, appears active even in death: sometimes survives the death of other functions, as seen in transudations standing in drops upon the skin: the steam or vapor arising from the body, charged with the febrile odor, is obvious to the sense of smelling.

The following inquiry relates not to the physiology, pathology or therapeutics of temperature, but to its morbid and *post-mortem* history. It is not a little remarkable, that the facts should be so few and so discrepant on this subject.

Will not the period arrive when the thermometer shall become the constant companion of the physician, and his guide as to certain remedial measures at the bed-side? Or is it a matter of indifference, in all diseases, whether the patient be plunged into a hot bath, or steamed nearly to scalding by the Thompsonian doctor, or macerated in cold water by means of cloths for hours, then plunged into a cold bath, to be followed with interminable douches and drinks? Must the patient during every walk, at every water-fall or pool, take a fresh bath, plunging and drinking like a fish, after the mode of the Silesian farmer, *Priessñitz*, and his many followers?

The Thompsonian declares that the only cure is heat; the Silesian, that the only cure is cold water? Why should a sick person, every where from 5° to 8° hotter than in health, be subjected to hot mustard baths, or another suffering from concentrations of internal heat, while the skin is in an algid collapse, be enveloped in the cold-water apparatus? Is there no criterion but caprice? If the physician has not the patience to call to his aid the thermometer in order to give precision to his prescriptions, let there be a Thermist or Themometer-man, or let the cupper add this instrument to his apparatus. At the present moment, while the cold-water treatment is spreading, it is the more necessary to seek precise knowledge upon this subject.

I now proceed to give an account of the circumstances attendant upon the following experiments:

Finding no thermometer exactly suited for my purpose I chose, as the least inconvenient, Reaumur's bath thermometer, having a double cylinder, one in the other; the outer one enclosing a paper scale. This, with all its tardiness, is the best I have yet seen; though I intend to direct the construction of two thermometers, so modified as to answer the purposes of the operator better than any I have used. It is evident that the common thermometer, with the metallic scale, is inapplicable; the stem is too fragile to be trusted to the hand of a delirious patient; the bulb being globular, or irregular, or too small, cannot be grasped accurately, or confined by a bandage in the hand so as to exclude the air. A very large instrument, containing much quicksilver, robs the part to which it is applied of too much heat. For the hand, the popliteal region, and the cavities, a rod-shaped bulb, about two inches long—and for the surface of the forehead, trunk and thigh, a flattened and curved bulb, would be most convenient.

The calorific power, susceptibility, and conduction of the body differ greatly at different times, without any very appreciable changes in the circumstances. Sometimes my instrument attained its maximum in 5, sometimes in 10, sometimes in 15 minutes. On some occasions I refrigerated the instrument with ice-water, but this I found not only erroneous in its result by robbing the part of its natural heat, but it prolonged the experiment to an inconvenient extent. On the contrary the instrument ought to be kept nearly to the degree of the part, though this I often omitted. I have tested the instrument I used by thermometers made in Paris, in London, and in this country; by the published tables of Mr. Lillie of this city, without finding any discrepancy worthy of attention; also by boiling water, which agreed exactly in the last two of four trials: in the summer two experiments fell a little short of 80° R., 212° Fah.

Comparison of my observations with R.'s Ther. from Jan. 9th, 1844, to the 16th of the same month, with those referred to above, taken with Fah's. and published in the Republican newspaper for the same period.

NEW ORLEANS, LAT. 29° 57′.

| Day of the month | 8 A. M. | | 2 P. M. | | 8 P. M. | |
|---|---|---|---|---|---|---|
| Jan. | Fah. | Reau. | Fah. | Reau. | Fah. | Reau. |
| 9 | 64 | 65 | 74 | 73 | 70 | 70½ |
| 10 | 70½ | 71 | 68 | 69 | 64½ | 64½ |
| 11 | 62 | 62½ | 64½ | 64½ | 63 | 65 |
| 12 | 69 | 70½ | 78 | 78 | 72 | 73 |
| 13 | 61 | 60 | 71 | | 61½ | 59 |
| 14 | 62 | 62 | 79 | 77 | 71 | 74 |
| 15 | 72 | 73 | 64½ | 65¾ | 63 | 64½ |
| 16 | 63½ | 65 | 58 | 58 | 49½ | 50 |

Though the bath thermometer, (*thermométre pour les Bains*), reaches its maximum usually in 5 to 10 minutes, yet this is not always the case, especially in cool weather, and in some cases after appearing to be stationary a short time it begins to mount slowly a degree or even more: I have known it to decline as much in a few instances without any very evident cause.

The principal points of demonstration in the living body are the following: The hand, the axilla, the folds of the groin, the perineum, and the back part of the knee joint, when the leg is bent so that the heel shall press against the thigh. The horny induration of the skin of the hand incidental to laborers forms no objection if the instrument be held properly. It is a remarkable fact that the patient, even when more or less delirious, is apt to hold the instrument in a perfect manner; he grasps it a little too strongly; he fixes his attention upon it and drops his incoherent conversations. In raving delirium and deep coma the operator's fingers must be applied so as to close the patient's hand, or a bandage, or a handkerchief may be used for the same purpose. When the instrument is applied in the axilla the patient by lying on his side compresses it effectually excluding the air: nearly the same effects may be attained in the perineum, and in the scroto-inguinal region by crossing or flexing the thighs. The popliteal region is one of the most convenient places, though unfortunately it did not occur to me in time to avail myself of it to any great extent: the patient lying on his back draws his heel against the buttock, or he may lie on his side and do the same, or the leg may be fixed against the thigh by carrying a single band around the ankle and upper part of the thigh. These points form for practical purposes a good, though not a perfect calorific circle, because the feet and head are excluded. The tongue so much relied on is for many reasons ineligible; breathing, moisture, evaporation modify the result; it is inconvenient and repulsive, and if the patient be delirious it is impossible.

In the dead subject these points, except the hand, are alike available, together with many others of greater value, as the rectum, vagina and artificial punctures, by which all the cavities may be explored with facility. These latter should be made no larger than is absolutely necessary to admit the instrument, say the third of an inch, and in such a manner as to exclude the air. This is easily done upon the chest, &c., by drawing the skin so that when punctured it shall act as a valve over the internal orifice. The highest portion of the pericardium and heart may be stabbed, so as to admit the instrument without exposure; the instrument may be forced into the parenchyma of the liver without any cutting in most cases.

For a considerable time I was at a loss how to reach the brain without exposure. All known modes of opening the brain are bad, nay sometimes murderous in a pathological sense; the saw cuts vessels in the dura mater or even deeper, causing sanguinous effusions and discolorations, discharging at the same time, the serosity in the arachnoidal sack; the hammer often produces the rupture of minute vessels at and under the point of the stroke. Even on the other side of the head, where the counter stroke (*contre-coup*), is received ecchymoses, and bloody infiltrations of the membranes of the brain may and often do thus happen.

At length it occurred to me that the orbit of the eye would afford every possible facility of reaching the brain without admitting the air. The tissues at the inner angle of the eye were divided—an iron punch as large as the thermometer was used to perforate the thin bones of the orbit which requires but little force—the thermometer passes readily, without even destroying the eye in most cases.

Though I have not restricted my observations to yellow fever, yet this is perhaps the best fundamental type, not only for the study of febrile pathology in general, but for the study of temperature, because it is the most acute fever known, and because the various tissues of the body are usually but little altered by remote secondary sub-lesions, of a general character, not directly connected with the disease. For example, the exterior of the body though much discolored, presents no bed sores, no emaciation—the muscles no paleness, softness, inelasticity—the cellular tissue no serosity—the organs no atrophy, &c. The original malady is not lost in the labyrinth of tedious, transforming, secondary affections.

The external caloricity of yellow fever, is, in the living body, usually greatest in its primary, less in its middle, and least in its closing stages, at such points of demonstration as are most easy of access, though these variations may or may not prevail in the central organs. These propositions will be proved hereafter.

With a zeal deserving all commendation experimenters have sacrificed hecatombs of the inferior animals. Scorning to retreat into the mazes of an *unexplained vitalism* for physiological and pathological explanations, they have boldly appealed to the physical sciences, to vivisections, and to diseased conditions artificially produced, for the establishment of the principles which govern the living body in a healthy and morbid state. Even when they fail in these difficult enterprises, they can say as a royal but defeated soldier said after an unfortunate battle—"we have lost all but our honor."

Of the essential nature of an isolated vitality we know nothing. Of its material manifestations we know something, but that something is modified by those laws which govern dead or inert matter, presenting a very complex combination which it is difficult to analyse so as to show which is strictly vital, and which exclusively physical.

But in these crusades against vital explanations, at the head of which stands that veteran of vivisectors, Magendie, is there not danger of running into the opposite extreme? Is not an experiment, when modified by the agonies of mortal vivisection, or a fatal poison introduced into the blood-vessels, more or less equivocal, unsatisfactory, nay fallacious, in its application to a natural disease, or a physiological action, or any law of natural philosophy? It is but too much like that judicial proof, in the criminal jurisprudence of a period not long since passed, called THE TORTURE, consisting of screws, wedges, wheels, pullies, dislocations, fractures, burnings, drawings, and quarterings, so contrived as to extract evidence, by which the victim sometimes confessed not only the truth, but a good deal more, accusing himself of sorcery, witchcraft, and other supernatural and impossible misdemeanors?

Surgery has taken advantage of this mode of inquiry, and with the best results. A better example could not be referred to than that recently given to the world by Professor Gross in his experimental and critical "Inquiry into the Nature and Treatment of Wounds of the Intestines." (Western Jour. Med., 1843). But the *proof by the torture* in physiology and pathology must not be too much relied on, even when applied by such an operator as the distinguished Magendie. Dogs are available in surgery, but except hydrophobia they do not suffer much in common with humanity, as fevers, &c. Now this cannot be applied to the mode of proceeding I have adopted in taking the temperature as soon as sensation ceases. If the thigh be 113° sometime, say one to three hours after death, what may not have been the heat of the central organs throughout the whole malady? The greatest objection which theorists can bring against this mode of studying fever in its literal sense, immediately after apparent death, is the fact, or supposed fact, that the heat, morbid and healthy, must cease along with the action of the heart, lungs, and senses. Though these may die simultaneously in a few cases, yet such is not the fact generally. But the objection shows the importance of the inquiry, since it implies that however great the *post-mortem* fever may be, the *ante-mortem* fever must have been greater, and if so, pathologists have greatly underrated the intensity of febrile heat. This mode may have its modifying circumstances too, but they are natural, not artificial.

In the history of the progress ot the medical sciences, and particularly of physiological experimenting, animal temperature has a conspicuous place: this is a salient point in Baron Cuvier's celebrated work (*Hist. des Prog. des Scien. Nat.*) In almost every weekly meeting of the French Academy of Science, this is a prominent theme, as may be seen in the ponderous quarto tomes (*Comptes Rendus*), annually published by that learned institution. Among its members, Becquerel and Breschet have distinguished themselves by researches upon this subject.

The healthy temperature is a matter of the utmost importance in determining what may be regarded as morbid heat; Broussais says, "the observing physician never will attempt to investigate morbid affections except by the lights of physiology" (Chron. Phleg. v. 2, p. 10). It may be well to examine briefly what the authorities say on these topics. Becquerel and Breschet represent the heat of the interior of the body to be 98.6° Fah. (Compt. Ren. tom. 6, p. 438)—Fordyce 97°, (on Fevers)—Müller, (Phys.) 100$\frac{3}{8}$° to 101$\frac{1}{4}$°—Dr. Beaumont, the best authority on the temperature of the stomach, says it gives an average of 100°, (Experiments on Martin). Copeland fixes human temperature from 96° to 100°, (Dict., art., cold). Müller says that the temperature is lower, the farther removed the point is from the centre—axilla 98°; loins 96$\frac{1}{2}$°; thigh 94°; leg 93° or 91°. Again, mouth and rectum 97.7° to 98.6°: he thinks there must be some error in several experiments of Dr. Jno. Davy, in which Dr. D. found the heat somewhat higher in the rectum than in the brain, (Phys. p. 94). Magendie (Phys. by Prof. Reveré) lays down the same doctrine: "the chest is the centre of animal heat, which there approaches 104°, while the limbs are 88° or 90°" (p. 94). Again: "those parts which are most distant from the heart or receive less blood are colder—the limbs are colder than the trunk," (Edit. 1844, p. 448). Prof. M. Edwards (Anat. and Phys., p. 154), holds the same doctrine. Now, it will be seen that febrile caloricity is not distributed, at least in the dead body, according to these laws, until a certain period after death, the period of refrigeration.

Whether morbid heat be simply an augmentation of animal heat, or some deleterious combination of it with solar and terrestrial caloric, latent and free, is foreign to our present purpose. It will be necessary to take a rapid glance at its character, in the only point of view in which it has been contemplated by authors with some attention, namely, in its range in the living body, principally during fevers. We shall perceive, hereafter, the strong and unexpected contrast afforded by the dead body.

Müller's estimate of the maximum morbid temperature is in some diseases as high as from 106° to 107° Fah. Horn, Fordyce, and Elliotson put the maximum in fever at 107°; Dunglison at 106°, (Phys. vol. 2, p. 200); Currie at 105°, one case only at 108°, (W. Philip, Fevers, vol. 1, p. 124); Colhoun at 105° (Greg. Prac., note, vol. 1, p. 45); Bouillaud says that the heat in typhoid varies from 33° to 41° cent. 91$\frac{2}{5}$° to 105$\frac{4}{5}$° Fah., being always more elevated than in health, (Dict. des Dict. de Méd., t. 8, p. 646, Paris, 1841). Magendie says that the temperature of the body never exceeds 40° cent., 104° Fah. (Lect. on the Blood, Bell's Edit. p. 236). Sir Astley Cooper, who quotes Jno. Hunter, denies that there is any increase of temperature in the interior of the body from internal inflammation, though upon the surface in external inflammation he admits a rise of 7°, that is as high as 90°, under the following circumstances:

"On the inside of the thigh, where a blister was applied, the thermometer rose to 90°, whilst the inside of the opposite thigh was only 83°; in another case it rose only 4°, but in every experiment the temperature was increased (Cooper's Lect., vol. 1, p. 22-'3). Hunter's opinion is based on operations upon brutes. Dr. Cheyne found the heat in fever to rise to 109° (Prof. Bartlett on Typhoid, p. 264). Prof. Watson limits febrile heat to 107° Fah. (Lect. Pract., p. 85, 1844).

The great influence of moderately prolonged cold upon the human body has been but little studied and less understood. Dr. Dunglison considers it as proved, that exposure to cold is incapable of depressing the temperature of the system lower than about 15° beneath the natural standard." (Phys. vol. 2, p. 195). When the whole body sinks to 78° or 79° death takes place: (ib. p. 213). M. LAURENT says the same, though he places the vital heat unusually low in the following passage:

"Lorsque la soustraction de la chaleur du corps humain est trop brusque et trop continue, sa température vital habituelle, qui est de 32° C. (87.6° Fah.), descend à 26° (67.8° Fah). Alors se manifeste le besoin d'un sommeil invincible; c'est la torpeur ou l'engourdissment, précurseur d'une mort produite, suivant M. Chaussat, par l'epuisement des forces nerveuses, et probablement aussi par l'action direct du froid sur le sang, qui se coagule dans les vaisseaux."—(Dict. de la Conversat., vol. 12, p. 345, Paris 1834).

Dr. Currie found the temperature of a man plunged into sea-water at 44° sink, in the course of a minute and a half after immersion, from 98° to 87°; and in other experiments as low as 85°, but never lower than 83°. It was always found, however, that in a few minutes, the heat approached its previous elevation. Similar experiments have been performed on other warm blooded animals. (Ibid. p. 191).

The following experiments, made with much care and copied without any picking or selection, appear so far as they go to overthrow these conclusions. Observations more numerous, and not confined to a single person, might be more satisfactory.

I may here remark, that while sitting in a room artificially warmed the hand holding the instrument was always turned from the fire, and rested upon a table covered with a woolen cloth. During the experiments the hand was never opened, the temperature was usually noted every minute, or every five minutes, though not always so mentioned in this paper, from a wish to be brief in details.

Jan. 16th, 7 A.M.—Room 65¾°; experiments 1 hour in bed; crossing the thighs 97¼°; perineum and scrotum 100°; pressing the sole of the foot against the internal maleolus 97¼°; axilla 100°; bend of the elbow 98°; left hand 98°. Left the bed, put on no coat, in 30′ the hand gave only 90¼°.

3¼ P. M. Room 68°, caloricity of the left hand, after a trial of 35 minutes, 98°; water at 61½° was applied repeatedly to the forehead for 37 minutes; during this time the heat of the hand fell 8°; the person well clothed. There can be no doubt that here the whole system was cooled 8°; the face became pale, afterwards flushed.

Jan. 17, 6¼ A. M.. Bed-room 46°; left hand not covered by the bed-clothes, in 23 minutes gave 92°; leaving the bed, 8 minutes, without dressing to 85°; partly dressed, 20 minutes, 80°. In a room with a fire at 59°, in 15 minutes, 75° (here a slight exposure to a moderate cold caused a fall of 17°); went into the open air at 46°; water at 38° was applied to the face and both arms at intervals for 11 minutes, the hand fell to 72°; breakfasted 15 minutes, repeating the cold occasionally, 70°; went into the room where the fire was, as above, 15 minutes declined to 67°! At 10 A. M., the left hand was 96°, clothed: room 66°, good fire. Rolled up the cuff, applied water at about 44° to the wrist with a sponge, &c., at intervals; in 40 minutes the heat fell to 80°, in 15 minutes to 74½°; cold now applied to the forehead only, 10 minutes, 70½°; cold now withdrawn; sat by the fire 15 minutes; hand 68°; 15 minutes 69°; 10 minutes 81°; 15 minutes 93°; 30 minutes 98°; 10 minutes 99°.

The refrigeration progressed as long as the experiments lasted: in the first case depressing the temperature, in 1 hour 24 minutes, from 92 to 67°, a difference of 25°; in the last case, occupying nearly the same length of time, the fall was 28°; in 15 minutes after the withdrawal of the cold, aided by a comfortable fire, the hand was still 3° below the point of departure, but in 40 minutes more rose 3° above it.

Jan. 18th, noon. Room 65¾°; coat off, caloricity of the left hand 97¼°; about 12 lbs. of water at 50° used for a foot bath; 5 minutes while undressing the hand fell to 95°; 5 minutes while using the foot bath, to 90½°; 15 minutes while exposed to the air, to 89°; 5 minutes without coat, to 88°.

Jan. 19th, 6 A. M. Room 54½°; caloricity of the left hand in bed 100°; arose, dressed partially, in 25 minutes, 90½°; applied water at 53° to the forehead, face, and opposite hand at intervals; sitting in a room at 62¼ deg., the back towards the fire, in 48 minutes the heat fell to 83 deg., and remained stationary 10 minutes, as long as the experiment lasted. No cold was applied to the left arm, which was clothed as usual, except the coat. The refrigeration was through the right hand and forehead, the instrument in the left hand, as in all the other experiments, was not changed after leaving bed.

9 P. M. Room 70 deg.; caloricity of the left hand 99 deg.; removed coat, vest, cravat, opened shirt collar, and in 15 minutes the heat fell to 94 deg.

Jan. 20th, 6 A. M. Bed-room 63¼ deg.; in bed, left hand uncovered 15 minutes 99 deg.; axilla 10 minutes 99½ deg.; inner and lower portion of the thighs 10 minutes 98½ deg.; groin, flexing the thigh upon the abdomen 5 minutes 101 deg.; popliteal region 5 minutes nearly 99 deg. Dressed, except coat and vest, 10 minutes 92 deg.; about 4 lbs. water at 63½ deg., applied at intervals with the right hand to the forehead and face, in 40 minutes the left hand declined to 83¾ deg., and was still falling when the experiment ended.

12 M. The caloricity of my left hand being 99 deg., the clothes on, sitting by a good fire, the room 68 deg., the right hand and wrist were immersed in one gallon of water at 63½ deg.; in 25 minutes the left hand fell to 97 deg.; in 15 minutes to 93 deg.; (cold being also applied to the forehead several times). The right hand now wiped dry received the thermometer at 93 deg.; in 10 minutes the mercury stood at 73 deg.; in 5 minutes rose to 74 deg.

Jan. 21st, 5 A. M. Room 68°; in bed, hand exposed, 10 minutes and 5 minutes, each observation gave 98½°; popliteal region 8 minutes 100 deg.; perineum 5 minutes 101 deg.; groin 5 minutes 100 deg.; elbow, at the fold, 5 minutes 99 deg.; left hand 5 minutes 100 deg.; instep and sole 99 deg.; by increasing the bed-clothes the left hand gave in 8 minutes 101 deg. Left the bed and sponged at intervals with 1 lb. water at 65¾ deg., in 15 minutes the hand was 97 deg.; friction with a towel 5 minutes, 95 deg.; 5 minutes while dressing 94½ deg.; 5 minutes 93 deg.; 10 minutes stationary and then began to rise. In 15 minutes the axilla was 99 deg.

Jan. 22d, 6 A. M. Room 68 deg.; perineum, groin, popliteal, and hand, each, 100 deg.; thighs, instep and sole 99 deg.; while dressing, and after, the left hand fell in 15 minutes to 97½ deg.; in 27 minutes (occasionally applying water at 68 deg. to the forehead) it gave 92 deg.

10 A. M. Air 68 deg.; sat in the open air of a porch after walking several squares, the weather growing warmer 1 deg. per hour, in 65 minutes the hand fell to 78 deg., and was still declining, being then 22 deg. less than when in bed 5 hours before.

It will be seen by some of the preceding experiments, that cold air and cold water even partially applied, in rooms with and without fire, cause a reduction of the temperature sometimes 33 or 34 deg., below the natural heat; this depressed temperature so far from disappearing in a few minutes continues for an indefinite period, probably involving the whole system as well as the accessible points upon the surface, as appears, perhaps, analogically during refrigeration from applying cold to the forehead, opposite arm, and to the feet while the rest of the body may be clothed comfortably. I felt no injurious effects from these refrigerations except, perhaps, a boil just above the knee. As hydropathists ascribe these affections, or at least eruptions, to cold applications, and as I have been entirely exempt from them heretofore, I think it proper to mention this fact. There can be no doubt that even a moderate cold steadily applied will produce a great and long-continued reduction of temperature, extending to the whole system, a fact, the pathological and therapeutic value of which, must sooner or later be appreciated in calorific excitements, irritations, congestions, and inflammations.

Magendie, with but little charity to vitalists, refers animal heat wholly to chemical changes in respiration, and says "that the blood in the living vessels is, in truth, almost as directly influenced by the temperature of the atmosphere as the mercury in the barometer."* (Lect. on the blood, p. 46). Without by any means adopting this conclusion to the fullest extent, I am inclined to think that the calorific oscillations of the body follow those of the surrounding media to a much greater extent than is generally supposed, and that in morbid augmentations of heat anti-calorifics may be so applied as to refrigerate with more or less accuracy and advantage down to the healthy standard.

And though our facts are too scanty and inaccurate to enable us to generalize with advantage, in all cases, yet I am far from thinking that the student of medicine will regard this subject as one merely speculative and devoid of useful applications.

Dr. Johnson (Med. Chir. Rev., p. 479, Oct., 1843), says, "It is now generally admitted that the temperature of animals derives its origin from a living principle; this principle, Prof. C.

*Chaussier considers *caloricity* to be "a primary vital property, ceasing at death—augmenting with every cause that excites the vital activity." (Dunglison's Phys., v. 2, p. 201.

Caldwell (N. York Jour. Med., Jan. 1844, p. 51), "believes to be a specific kind of *imponderable matter*, as indispensable to vitality as carbon is to carbonic acid. The *materia vitae* is an essential element of living organized matter." It will be as difficult to account for the increase of heat after death upon this principle, as upon those principles advanced by Liebig, as respiration, digestion, &c.

Dr. Double, of France, and some others, say that the animal heat is depressed below the ordinary standard during sleep and in the morning. This latter so far as I have experimented is not accurate, unless by morning we understand the next hour after rising from bed. *A priori*, it might be expected that caloricity would accumulate in the body while lying enveloped in non-conductors, as blankets, and the motionless air, almost hermetically sealed by the bed-cloths during a winter's night. And such appears to be the fact. I have already offered several observations, much abridged, made in bed with both Fahrenheit's and Rèaumur's thermometers, showing the caloricity of the body under these circumstances. It appears from these experiments and the following that in 9 observations, made in bed, upon the hand, lasting a quarter of an hour each, on 11 consecutive days in January, the minimum was 92 deg., the maximum 100 deg., the average about 98½ deg., very nearly the same as the elbow, ankle, sole, instep, and thighs, the axilla being about 1 deg. higher; the perineal, scrotal, inguinal, and popliteal regions, from 99 to 101 deg.,—averaging, in 10 observations, 100⅕ deg.

Jan. 23d, 6½ A. M. Room 74 deg., in bed; hand, instep, sole, ankle, lower portion of the thighs, axillary, inguinal, perineal and popliteal regions, each 100 deg. At 9½ A. M., room 71 deg.; hand in 20 minutes gave 88½ deg.; at 11 A. M., room, 72 deg., hand in 21 minutes gave 95 deg.; at 9 P. M., room 75 deg., the hand in 15 minutes gave 99 deg.; the groin and axilla the same.

Jan. 24th, 7 A. M. Room 72 deg.; in bed, hand and axilla 100 deg.; groin, instep, thighs, elbow, and tongue, 98 deg.

each. At 9 A. M., room 74 deg.; hand in 15 minutes 96 deg. The maximum temperature of the room in which the experiments took place was 77 deg., the minimum 46 deg., the average about 65 deg.

I now offer a table, choosing 10 consecutive days, in which the records have been kept with the greatest accuracy, and with the fewest interruptions, showing the caloricity of the hand in the morning from 7 to 10; at noon, at 3 and at 9 P. M., with the temperature of the room, and that of the open air. During this period few if any appreciable circumstances, excepting those which are recorded, intervened to modify the results compared with each other. The weather was very cloudy, and the rains frequent and copious. The minimum temperature was 39½ deg., the maximum 79½ deg, the winds variable, with occasional thunder; fogs prevailed to an unusual extent.

OBSERVATIONS ON THE CALORICITY OF THE HAND.

| | 7 to 10 A. M. | | M. | | 3, P. M. | | 9, P. M. | | |
|---|---|---|---|---|---|---|---|---|---|
| January. | Temperature of the room. | Caloricity of the hand in 15 min. | Temperature of the room. | Caloricity of the hand in 15 min. | Temperature of the room. | Caloricity of the hand in 15 min. | Temperature of the room. | Caloricity of the hand in 15 min. | Weather, &c. |
| 7 | 61 | 84 | 68 | 100 | 67 | 100 | | | Fog—rain. |
| 8 | 63½ | 82½ | | | | 96 | 70 | 97½ | Rain. |
| 9 | 63½ | 90 | 69 | 96 | 70¼ | 96 | 74½ | 99¼ | Foggy. |
| 10 | 70¼ | 94½ | 74½ | 91½ | 75½ | 90½ | 74½ | 98½ | Rain. |
| 11 | 68 | 94 | | | 71¼ | | 74½ | 98½ | Rain. |
| 12 | 70¼ | 92 | 74½ | 100 | 74½ | 100 | 74½ | 98½ | Cloudy—rain. |
| 13 | 70¼ | 88½ | | | | | 72½ | 98½ | Light clouds. |
| 14 | | 94 | 73½ | 100 | | 100 | 77 | 100 | Cloudy—mosquitoes. |
| 15 | 73½ | 94 | 74 | 99 | | 99 | 74½ | 100 | Rain—thunder. |
| 16 | 72 | 92 | 72½ | 97½ | 71½ | 97½ | 72½ | 99¼ | Fog—rain—thunder—mosquitoes. |

This table affords upon analysis the following results:

Average of the morning, from 7 to 10 o'clock, - 90.15°
Average at noon, - - - - - - - - 97.71°
Average at 3 P. M., - - - - - - - 97.15°
Average at 9 P. M., - - - - - - - 99.00°
Average at 6 A. M., in bed, - - - - - 98.45°

I copy the following data on the caloricity of the hand at about 9 P. M., commencing at the termination of the preceding table, making a series of nineteen consecutive days, affording an average with a fraction (⅝°) over the preceding, but confirming the general result in a striking manner. Each observation lasted at least a quarter of an hour, and was noted with all possible exactitude.

It will be seen that the average caloricity of the hand in a room with a good fire at 9 P. M., does not differ materially from the average in bed, in the morning at 6 A. M. in a room without fire, and that in a few hours after leaving bed the temperature declines about 9°, but rises again by noon to 97.71°, rather declining than augmenting at 3 P. M.; at 9 P. M. it rises about 2° more.

CALORICITY OF THE HAND AT ABOUT NINE P. M.

| 9 P. M. | | | |
|---|---|---|---|
| Jan., 1844. | Hand—fifteen minutes. | Temperature of the room. | WEATHER, &c. |
| 17 | 98½ | 65¾ | Clear. |
| 18 | 97½ | 68 | Clear. |
| 19 | 98½ | 70 | Light clouds. |
| 20 | 98 | 72½ | Fog—rain. |
| 21* | 100 | 72½ | Fogs—clouds—mosquitoes. |
| 22 | 99 | 74½ | Cloudy—mosquitoes. |
| 23 | 99 | 75½ | Clouds—mosquitoes. |
| 24 | 96½ | 74½ | Thin clouds—mosquitoes. |

These imperfect statistics of caloricity constitute only a

* In New Orleans the minimum temperature of the 21st January was 50½°, the maximum 75½°, while at Montpelier, Vermont, the mercury fell, according to the Montpelier Watchman, to 40° below zero: a difference of more than 90 degrees. On the 6th January the mercury, near Montreal, was at 40° below zero, while at 8 o'clock at night the mercury was 94° higher at New Orleans. On the 12th January the mercury was reported to be 12° below 0 at Quebec, while at New Orleans, at 8 P. M., the mercury stood at 72½ above 0, or 84½° higher part of my observations on this subject; they have been made on but a single person, and are, I admit, too scanty for safe conclusions. My observations upon morbid and post mortem caloricity are of a very different character, occupying several months of arduous investigation on many different persons, living and dead.

The hard working student will soon find after bidding adieu to the lecture rooms of his teachers that much is unknown, much unsettled, upon many of the simplest points in physiology. Even the temperature of the hand is involved in discrepancies so numerous as to show the imperfections of that science.

Much attention has been bestowed upon Meteorology, particularly by the learned Dr. Samuel Forry, whose work stands in our country without a rival. Let the same pains be taken to ascertain the laws of human temperature, healthy and morbid, at different periods of the seasons and of the day—and in different maladies in all their stages when modified and unmodified by remedial agents, and in all accessible points, and the greatest advantages will probably result.*

This imperfection in the physiology and pathology of animal caloricity, is, unfortunately for the student, but too well expressed in the therapeutics of heat, as laid down by the most eminent authors. Cruveilhier gives *enemata* in cholera, at 36°, Réau.=113° Fah., (*Anat. Path. livr.* XIV.) In the *Dict. des Scien. Méd.*, under the head *Du bain d'eau trés chaude* it is said: *il est trés chaud vers* 34° *R.*, nearly 109° F. Under the same terms, Prof. Hatin, of Paris, (*Méd.Opérat.* p. 73,) gives the hot bath at the temperature of 36° to 40° R.,—113°

* The difficulty, perhaps impossibility, of tracing any absolute connexion between the meteorology of the seasons and their epidemics, is set forth in the following passage:

"Pendant 10 années consécutives le Rapporteur de la Commission a publié trimestre par trimestre; l'histoire des maladies regnantes á Paris rapprochées des caracteres des saisons dominantes: il doit á la vèrité de déclarer que si ce travail ne lui a que peu serie pour remonter aux causes des grandes épedémies, il lui a éte très utile pour le divageur dans ses exercises cliniques.—*Comptes Rendus, tome 9im. p.* 515; 1839 *q. Rapport par M. M. Arago et Double.*

to 122° Fah. Yet, Laurent, (*Dict. de la conversat.* v. 12, p. 345, Paris, 1834,) says that a temperature of 104° Fah. can be supported only a few moments without danger: "L'application des corps solides chauffés jusqu'—40° cent. (104° F.) ne peut être supportée quelques instants sans danger." Dr. J. Bell fixes the hot bath at and above 98°; at that point the line of agreeable warmth is past. (*Baths and Min. Wat.*) De La Roche could not support above ten minutes and a half a *vapor bath*, which at first at 96.5° Fah., rose in eight minutes to 124.45°, and afterwards fell one degree. Berger was obliged in 12½ minutes to come out of a vapor bath, the temperature of which had risen from 106.25° Fah. to 128.75°. He was weak, and tottered on his legs, and was affected with vertigo.

In water a still lower temperature becomes insupportable. Lemonnier, being at Bareges, plunged into the hottest spring, which was 113° Fah. He could not remain in it above eight minutes. Violent agitation and giddiness forced him out. (Trans. Jour. Med., v. 9, p. 547). Let these facts be borne in mind. We shall see in the thigh a *post-mortem* heat which is here pronounced insupportable even for a few minutes.

Copeland (Dict Med., art. Cold), says, that man cannot exist for any considerable time in a mean range of temperature above that of his own body. In no part of the globe is the mean annual range of atmospheric heat within twelve degrees so great as that of the living frame.

Morbid caloricity it would seem may augment to such a degree as to cause the combustion of the body. This curious, and apparently well authenticated malady is, in some cases, not suddenly fatal, being local or partial in its action, as was the case with the Priest Bertholi, who lingered to the fourth day. A considerable number of fatal cases of spontaneous combustion have been reported by the physicians and by the judicial officers of different nations, as may be seen in the periodicals and medical encyclopedias. Prof. Short, of Louisville, Ky., reported a case a few years since in the Trans. Med. Jour., v. 3, p. 142. Partington, in his British Ency.,

(London, 1835), v. 1, p. 346 *et. seq.*, reports many: among them, the cases of the Countess Cornelia Bandini, Grace Pitt, Mary Clues, Jean Millet's wife, Mary Jauffret, widow of Nicholas Gravier, Mdlle Thaurs, a woman in Massachusetts, (16th March, 1802), a case from the Transac. Soc. Copenhagen, with other circumstantial histories.

If we admit the possibility of open combustion in the living body, can we hesitate to allow the possibility of an imperfect, smouldering, semi-combustion, or, at the least, a morbid heat, in the tissues during intense fevers? Magendie, not less distinguished for his physiological researches, than for his bitter denunciations of pathology, "that science which," according to him, "does not yet even exist," asks with apparent triumph, "what sense, in truth, is there in applying the word inflammation to our organs? Do our tissues really take fire? I confess I know of no example of such a phenomenon. When the blood rushes to a part in abundance, a certain rise of temperature, no doubt, occasionally follows; but it only reaches but a few degrees above the normal standard of the organ and never exceeds that of the blood in the left ventricle." (Lect. on the Blood, p. 32.) Now, as he refers heat solely to the chemistry of respiration which raises the blood only 1°, the left ventricle is therefore only 1° hotter than other parts. (Vide Phys).

At one moment Magendie declares that the temperature of the body depends upon the passage of the blood through the tissues, and at the next, he ascribes the heat in inflammation "to the accumulation or arrestation of the blood in the part!" (p. 25). Plain contradiction! So of the lungs. They make the heat, and yet they make the cold too, whenever it is necessary, as when a man enters a red-hot furnace. He expressly says that they are "an admirable cooling apparatus," and yet in his Physiology he says they are "the centre from which animal heat is developed." The histories of morbid and post-mortem temperature, which will be given, will show whether these assertions are stronger proofs than facts themselves, even though unconnected with theoretical fortifi cations.

It is proper to state that no special theory of fever will be attempted in this paper. It is not pretended that mere heat is alone sufficient to explain all the phenomena of fever.

With respect to post-mortem fever, which is perhaps nearly uniform, to a greater or less degree, let it be observed that circumstances, not necessary to mention, often prevented me from ascertaining the febrile caloricity until after it had declined more or less, so that the body had already felt the influence of the surrounding media. The morbid caloric may in some instances flow from the centre outwardly for a time more or less prolonged. But when it begins to leave the limbs it falls with great rapidity under the dominion of the atmosphere: the centre then becomes comparatively much the hottest, until near the commencement of putrefaction, at which time the entire body nearly coincides with the atmospheric temperature. In rare cases, where much blood has been lost, especially by the lancet, the heat augments but little, scarcely as high as in health, though probably higher than it was some hours before death; in these cases, muscular contractility is usually feeble and transient; rigidity is tardy and imperfect.

The doctrine of a post-mostem fever, or morbid heat, if sustained by facts, deserves neither sarcasm nor ridicule. Will any one pretend that respiration, or arterial circulation, or the nervous system acts with increased power after death? Yet, theorists ascribe animal heat to one or all of them with the confidence of a mathematical truth, in defiance of the antagonism of facts. The admirable Bichat says: "To derive general principles from facts—who are we, that we should turn away from this path?"

Broussais, who upbraids the ancients for "considering fever as an unnatural heat, (Path., p. 110), seems to ascribe to its physiolcgical action an omnipotent power. Caloric is the first and most important of all stimulants; if it ceases to animate the economy, others lose their influence over it; all the preservation, recuperative, and sanative phenomena cease. Caloric brings into play the unknown power, which constructs the organs." (Prop. ii, iv, v, Path). Now, in his first pathological proposition, he says, "health is the regular, disease the irregular exercise of the functions," and, of course, any great irregularity in the calorific condition must constitute disease in the same degree.

Among the preceding authorities the discrepancies as to temperature are sufficiently discouraging, but many of them probably grow out of the fact that the exact modes of investigation have not been identical. In making an experiment, all the modifying circumstances, as well as each step of the operation, ought to be attended to and made known. For example, let us suppose that the air is 40°; the thermometer at this temperature is taken into the hand at 109°; or, suppose that it is introduced into the middle of the thigh at 113°: here the parts in contact with the thermometer are necessarily robbed of much heat; 69° to 73° must be given to the instrument to be replaced, slowly, by heat conducted from, perhaps, the whole mass of caloric. In the experiments upon post-mortem caloricity, I have generally guarded against such errors by taking sufficient time. I may have erred occasionally by neglecting to prolong the experiment sufficiently.

Some account of the circumstances under which this inquiry upon animal temperature began may not be improper. Although the heat of the dead often struck me as remarkable, yet I did not suppose that it increased after death, until, during the last year, I determined to turn my attention to the following question: What are the most certain signs of death? Among many things, heat presented itself as one element worthy of investigation. This inquiry became the more interesting in consequence of certain acts of the Board of Administrators of the Charity Hospital in the summer of the year 1843. Upon this subject a little digression may be proper. Among other things, a rule passed forbidding post-mortem examinations, until six hours after death. The Board consisted of distinguished citizens; fortunately for the cause of science, they did not enforce this rule; neither the faculty nor the public seemed to think it necessary. The New Orleans public, ever charitable to the living, cares not to introduce, or patronise, absurd prejudices about the dead,—prejudices which elsewhere have trammeled the progress of the healing art. Common sense, in spite of any speculation, finds no difficulty in deciding that the fever subject, after undergoing the fatal symptoms, under the eyes of the physician and others—after ceasing to breathe, &c., is dead. Thought, sensation, pulsation, locomotion, every possible relation to the external universe, and existence itself, are no more. In swooning, asphyxia, hanging, drowning, epilepsy, &c., doubts may sometimes exist. If the living body be committed wholly to the charge of the physician, why not the dead?

The surgeon, who mutilates for the mere *eclat* incidental to operations,—the physician, who needlessly subjects his patient to hazardous medications,—the pathologist, who would proceed to *post-mortem examinations*, with even a doubt upon his mind whether the subject was dead, in the common acceptation of the term, would deserve the utmost execration of mankind.

The frightful stories of Bruhier, (*Dict. des Scien. Med.* t. 25, p. 174,) seem to have long terrified both the public and the faculty in the old world. He reports 180 cases of persons reputed dead, who were not so: 60 interred alive; 40 opened before death; 53 returned spontaneously to life, after having been laid out in their winding sheets; 72 reported dead, were alive. These, and similar statements, have caused some benevolent individuals to leave annual prizes to those who should discover the most certain signs of death, with a view to prevent premature interments.

The more this topic has been elaborated by learned men, the less unanimity appears to have resulted. The unlearned nurse readily recognizes the physiognomy, and other signs of death, though perhaps unable to describe them with certainty. No one, it is believed, after apparent death from fever, in New Orleans, has ever been known to return to life: this, practically speaking, is death. The French Academy records a marvellous case, noticed by Baron Larrey, in which a French officer, (M. Morel,) then living, was taken for dead in two instances, and buried as such. "M. Julia Fontenelle transmet une observation tirée du cinquième volume de la clinique de M. Larrey et la quelle il résult qû un officier français (M. Morel) encore vivant á été doux fois enterré comme tel. (Com. Ren. T. 2, p. 257, Mai 1836.) The Academy occupies itself much upon this subject: it has been appointed to award (décerner) prizes in relation to its elucidation, but appears to incline to the test by putrefaction!

M. Donné, in a letter to the Academy upon the signs of death drawn from the alterations of the globules of the blood, remarks, that "tout en reconnaisant, que le signe le plus certain de la mort, le seul que soit á l'abri de toute erreur dans les cas douteux, est la putréfaction." (*Com. Rend.* T. 6. p. 345.)

Some of these *savans*, not satisfied with a delay of twenty-four hours as fixed by the French Code, wish the period protracted: "M. de Brière écrit relativement aux malheurs qui peuvent résulter des inhumations trop precipitées. Il désizerait que le *délai de quarante-huit heures*, dit-il, a lieu en Angleterre et Bavière, fut adopté en France." (*Ibid.*)

Lastly, a medical writer, in Paris, during the year 1843, insists on putrefaction,—greenness of the tissues, &c., as the only certain tests, requiring about three days! But in the steady cold of winter, this might require as many weeks before greenness, &c., would appear.

If putrefaction be regarded as the only certain test of death, then, adieu to all confidence in post mortem examinations, as the means of ascertaining morbid appearances and of guiding to sound pathological deductions. Magendie, who sometimes is quite skeptical, says, "I set but little value on minute examination of the traces left by disease upon organs, though that pursuit has been pompously styled pathological anatomy: are you not in truth convinced that the lesions found at our autopsies are frequently produced after death, and that consequently the plan hitherto followed in such inquiries is fallacious, and can only lead to vague information and error?" (Blood, p. 276.)

The February No. of this Journal, for 1843, containing a part of my observations upon the fallacious results incidental to late dissections, was sent to one of the most illustrious pathologists in Europe, Louis, of Paris. In a letter which I had the honor to receive from him since, he speaks upon this subject in the most decided language. This testimony is not only of the greatest weight in itself, but displays a candor, a love of truth, which an author wholly influenced by sordid self-interest would neither feel nor exhibit, since some of M. L.'s own distinguished works have been based on dissections more or less tardy. Dr. Louis says:

"Au sujet de L'anatomie pathologique, vous avez fait sans doute ce qu aucun médicin nã fait jusqú ici; une il úa été donné â personne á part quelques circonstances infiniment rares, de faire des autopsies quelques minutes seulement après la mort, quand la roideur cadavérique (une des signes le plus certains de la mort) n'existe pas encore â beaucoup par; vous avez vous pu voir ce que peu de personnes ont vu, constater des lésions susceptibles de s'effaçer promptement; et si vous avez pu receuillir très en détail, et jusqu'â la dernière heure des malades, les symptomes qú ils eprouvés vous feriez une chose très utile â la science de publien ce que vous avez vu."*

From the above extract, it would seem that Dr. Louis regards cadaveric rigidity (*rigor mortis*) as one of the most certain signs of death.

At the present time, the medical faculty in New Orleans appear to be unanimous in the opinion that late dissections must

---

* "As to the subject of pathological anatomy, you have done, without doubt, that which no other physician has done till now; for it has been given to no person, to make post mortem examinations a few minutes only after death, when cadaveric rigidity (one of the most certain signs of death,) exists as yet to no great degree. You have been able to see that which few persons have seen,—to establish lesions susceptible of changing quickly; and, if you have been able to collect in detail, and until the last hour of the patients, the symptoms which they have experienced, you will do a thing very useful to science to publish what you have seen."

lead to results more or less fallacious, and, some go so far as to declare that a dissection, after even six hours, is more likely to mislead than to instruct. New Orleans, happily, is free from those prejudices, which in some parts of the Union punish as a felony the taking of the dead for examination—a punishment seldom inflicted for stabbing, shooting, or otherwise slaying the living, in these days of legal ingenuity. At one of the first dissections (after the act of the board), where the body had been kept six hours, the medical gentlemen abandoned the examination, unfinished, on account of the obscurity cast on it by post-mortem discolorations; a learned anatomist, now no more, was present, and expressed the same views—this was Dr. Harlan. A few weeks after, in the midst of the labors incidental to the epidemic yellow fever, he fell suddenly with apoplectic symptoms, and so little did *he* fear premature dissection, that he requested (as I was informed) that his body should be examined in ten minutes after death.

It was, therefore, during my researches upon the signs of death, (the results of which I may publish hereafter), that I accidentally ascertained the existence of a post-mortem fever, or caloricity. I then turned my attention to the calorific history of the living body, covering the whole ground from the second hour of the fever to the period of putrefaction. The symptoms and treatment of those who recovered, and of those who died, including post-mortem examinations, I will be obliged to omit and to restrict myself to temperature alone, hoping that these observations, however imperfect, will contribute something to fill up an evident gap in febrile pathology. Whether pathology shall settle down into solidism, or run into fluidism—whether it shall take the symptomatic or idiopathic, the chemical, mechanical, or modified vital form, one thing is certain, the *positive* alone should guide us. Caloric is the great motor of the universe. Its pathological relations, combinations, and deteriorations, solar, terrestrial and animal, ought to be studied in preference to hypothetical agents, unknown gases, or vital metaphysics. It is no aggression—no violation of the sanctuary of vitalism, to take from it any of its supposed holy vessels, which may of right be claimed by the laws of simple physics or chemistry. It is often a clear gain to the cause of truth when any supposed vital law is shown to be identical with those of mechanics, hydrostatics, pneumatics, electricity, caloric, or chemistry.

As to the supposed good effects of febrile heat, compared with coolness of the skin, noticed by Dr. Chyene, (Bartlett's Typ. Fever, p. 264—'5), the circumstances, the stage of the disease, &c., are not given with sufficient particularity. Heat, like the blood, may have a congestive character; the skin may be colder than natural, while the heat is centralized even in algid cholera, in which the skin is icy, while the patient complains of the most intense burning heat inwardly. Prof. Cruveilhier found, when about to make his autopsies, in cholera subjects, that they were hotter (how much he does not say) than during life—"Froid beaucoup moins prononcé que pendant la vie." (Anat. Path. Livr. xiv. p. 26.) As illustrative of this subject, and the origin of this investigation as mentioned above, I will here recount a part of my observations, in the first case in which I took the post-mortem temperature. The heat, in this case, was less than in many others—the history is less perfect—no positive thermometrical data had been recorded before death. The skin, during the two last days of the malady, was so remarkably cold, that some medical gentlemen regarded the case as congestive, rather than yellow fever; but the history, as well as the autopsy, fully satisfied me that it was the latter. The stomach contained 12 ounces of black vomit.

A native of the United States, born about the 40th degree N. latitude, aged 25, late a steamboat man in southern Louisiana, resident in New Orleans two weeks; died of yellow fever Aug. 7th, 11 A. M., 1843, after an illness of four days and a half.

Air of the dead house 83°. Experiments began 20 minutes after death: axilla, 5′ 108°; under the tongue, 5′ 103°; epigastrium, 5′ 108°; 5′ same; perineum simply closing the limbs, 10′ 104°; centre of the thigh, 10′ 109°; tongue, 10′

100°; epigastrium, 5′ 109°; axilla, 5′ 104°; thigh, 5′, old incision which had been made too large, admitting the air freely, 107°; 3 successive obs. in different parts of the abdominal cavity, each lasting 5′, gave nearly 107°. Experiments now ceased 1 hour and 35 minutes; resumed 3 hours and 20 minutes after death. Dead house, 81°; epigast., 10′ 104°; outer side of the lungs, 10′ 103°; base of the right lung, 5′ 104°; between the liver and diaphragm, 4 hours after death, 104°. Temperature of the brain not taken. The body rested on a stone floor, which was placed directly on the ground—no cover but a linen sheet, which was removed when the experiments began; the room much ventilated—brisk winds, with heavy showers of rain, during the observations.

In this case the coolness so remarkable to the touch disappeared in 15 minutes after death. The axilla at 25′, and the epigastrium at 40′, both gave 108°, while the thigh, 1 hour after death, gave 109°; in 1 hour 15 minutes the epigastrium attained the same elevation as the thigh. This, the culminating era of the morbid caloricity, was followed by a gradual but slow declination, differing much from simple atmospheric refrigeration, inasmuch as the thigh and the epigastrium, and other abdominal regions, after trials for twenty-five minutes, gave exactly the same temperature. Refrigeration from the surrounding *media* progresses from the circumference to the centre, until the putrefactive period, when the air, the surface, and the centres, coincide very nearly. The body exposed to a hurried, brisk wind, at 81°, for a period of four hours, gave in the centres, 104°—that is, 4 or 5° beyond the healthy heat.

I have already copied from my MS. volumes, 162 calorific histories, which, with a few more made during the present winter, (1843—'4), will complete the series. I own I am not without some fears of making a premature publication, as this inquiry has occupied me for a much shorter period than any other great branch of febrile pathology. And, if one so humble might be allowed to apply to his own case, the words of so distinguished a man as Sir Benj. Brodie, I would say, "that one principal result of my labors has been to convince me that life is not long enough for these difficult researches; that the utmost which can be accomplished by the zeal and industry of an individual is to make such progress in the study of pathology as may enable those who may come after him to carry their inquiries farther; and that the expectations of any one who aims at higher objects than these must terminate in disappointment." (Am. Jour., Oct. 1843.)

Few, except those who have gone over the same ground, can imagine the labor and irksomeness of these dismal researches along the frontiers of death. Silent, alone, sitting for hours on a coffin, among dead bodies bearing on their saddened faces the impress of the last agony, watching the wanings of a lingering vitality and the steady advances of the great annihilator, decomposition, with scalpel, pencil, book, and thermometer, like Sterne's prisoner in the dungeon, who etched down with a nail his diurnal history—these are circumstances that fiction cannot heighten; pursuits that honest Charon, the ferryman to ghosts, upon the dark and polluted Styx, would scarcely undertake. The fabled shades, Sorrow, Fear, Sleep, Night, Sickness, and Death, and the Valley of tears, might very well be located in and round about hospitals and dead houses.* The moral associations they fix upon the minds of their inmates are often unfavorable in a medical point of view.

---

*Eugene Sue, the celebrated novelist, in *The Mysteries of Paris*, has drawn a dark, but too true a picture, of the moral, mental, and medical influences which reign in the wards of a crowded hospital. The shame, disgust, and inconvenience, (especially to the female patient), incidental to interrogations and examinations, repeated at pleasure, by physicians and students, before company, by day and by night, have not been at all exaggerated: to these have been added, the fear of dissection after death,—a fear not the *less real* because it is *absurd*: "Ah! it is horrible! said Clèmence, shuddering with affright. One must come here [to the hospital] to know that there are, for the poor, misery and alarms even beyond the tomb." Sue makes his scientific Dr .Griffon too fond of experiments upon the poor,—too brutal in his speeches before his patients, upon the fine morbid specimens that their bodies will afford, and, in directing his student to cut the initials of his name upon the corpse of the actress in the presence of the sick, preparatory to dissection.

Romance, imagination, reason, nay, science itself, "serve but to light the troubled way," giving a greater force to the bitter realities, always transpiring before our eyes, in these institutions, and at the bed side. However wise it may be in the multitude to repel such ideas by gay illusions, the physician, from his avocations, cannot long enjoy them. He will often think in the following strain, with a veteran medical journalist—a passage, by the way, very much like one I have seen in some of Voltaire's works: "Man is the only animal in existence who knows or even thinks he must die. This piece of knowledge seems to be a necessary accompaniment of Reason, and no small drawback upon the pleasures of that boasted gift! It is a prescience which embitters a great portion of the life of man! On which side does happiness predominate? On that of man or that of beasts? Those who have lived longest in this world and seen most of human nature, will have little hesitation in casting the balance in favor of animals. The young and the sentimental will come to a different conclusion. The knowledge of literature, arts, sciences, so far as happiness is concerned, is rather a curse than a blessing." (Med. Chir. Rev., Jan. 1843). I make no apology for thus "heaving a moralizing," if not a scientific sigh, upon this subject—the affectation will be found on the side of levity and insensibility. But leaving the mental pathology, I will in the concluding part of this essay, confine myself to the physical, the positive alone. Here, if anywhere, the Genius of Pathology, like the dove which returned to the Ark, finds rest for the sole of her foot, and seeks among the dead that which may be useful to the living.

New Orleans, March 30th, 1844.

[TO BE CONTINUED.]

